# The role of conceptual knowledge in understanding synaesthesia: Evaluating contemporary findings from a “hub-and-spokes” perspective

**DOI:** 10.3389/fpsyg.2014.00105

**Published:** 2014-02-19

**Authors:** Rocco Chiou, Anina N. Rich

**Affiliations:** ^1^Department of Cognitive Science, Macquarie UniversitySydney, NSW, Australia; ^2^Perception in Action Research Centre, Faculty of Human Sciences, Macquarie UniversitySydney, NSW, Australia; ^3^ARC Centre of Excellence in Cognition and its Disorders, Macquarie UniversitySydney, NSW, Australia

**Keywords:** synesthesia, multisensory integration, attention, color memory, object knowledge

## Abstract

Synesthesia is a phenomenon in which stimulation in one sensory modality triggers involuntary experiences typically not associated with that stimulation. Inducing stimuli (inducers) and synesthetic experiences (concurrents) may occur within the same modality (e.g., seeing colors while reading achromatic text) or span across different modalities (e.g., tasting flavors while listening to music). Although there has been considerable progress over the last decade in understanding the cognitive and neural mechanisms of synesthesia, the focus of current neurocognitive models of synesthesia does not encompass many crucial psychophysical characteristics documented in behavioral research. Prominent theories of the neurophysiological basis of synesthesia construe it as a perceptual phenomenon and hence focus primarily on the modality-specific brain regions for perception. Many behavioral studies, however, suggest an essential role for conceptual-level information in synesthesia. For example, there is evidence that synesthetic experience arises subsequent to identification of an inducing stimulus, differs substantially from real perceptual events, can be akin to perceptual memory, and is susceptible to lexical/semantic contexts. These data suggest that neural mechanisms lying beyond the realm of the perceptual cortex (especially the visual system), such as regions subserving conceptual knowledge, may play pivotal roles in the neural architecture of synesthesia. Here we discuss the significance of non-perceptual mechanisms that call for a re-evaluation of the emphasis on synesthesia as a perceptual phenomenon. We also review recent studies which hint that some aspects of synesthesia resemble our general conceptual knowledge for object attributes, at both psychophysical and neural levels. We then present a conceptual-mediation model of synesthesia in which the inducer and concurrent are linked within a conceptual-level representation. This “inducer-to-concurrent” nexus is maintained within a supramodal “hub,” while the subjective (bodily) experience of its resultant concurrent (e.g., a color) may then require activation of “spokes” in the perception-related cortices. This hypothesized “hub-and-spoke” structure would engage a distributed network of cortical regions and may account for the full breadth of this intriguing phenomenon.

## INTRODUCTION

Although subjective experiences (qualia) vary across individuals, the majority of people seem to perceive the world in a similar way, providing a common ground for communication. For example, we can stand together overlooking the ocean and agree that the water appears to be a shade of blue, we can discuss elements of a lawn being green and brown, and so on. For individuals with *synesthesia*, however, the experience of the environment can be quite different from the rest of us. A synesthete may experience the flavors of different food when reading words in a sentence, or different colors when thinking of the letters of the alphabet. The triggering stimulus (inducer) and resultant experience (concurrent) can be in different sensory modalities (e.g., listening to music elicits a taste), or within the same modality (e.g., reading achromatic text elicits colors associated with each word or letter). Estimates of the prevalence of synesthesia vary considerably, depending on the definition of synesthesia and the sampling method. If we look specifically at grapheme–color synesthesia (graphemic stimuli, such as letters, digits, the names of days or months and other words, trigger colors), estimates vary between ~0.08% (Rich et al., 2005) and ~2.8% of the general population (Simner et al., 2006; Banissy et al., 2009). Simner et al. (2006) define day-color synesthesia differently from grapheme–color synesthesia, and estimate it occurs in up to 4% of the population, whereas Rich et al. (2005) consider individuals with only colored days as having a variant of grapheme–color synesthesia. Other types of synesthesia (e.g., music induces colors) are less common than grapheme–color synesthesia (for discussion, see Ward, 2013). Synesthesia has become a “hot topic” in cognitive neuroscience. Although there is an intrinsic fascination with phenomena that reflect extraordinary subjective experiences, this focus on synesthesia also reflects the potential that synesthesia has to provide insights into “normal” mechanisms of cognition and perception.

The primary questions addressed by synesthesia research to date are the level at which an inducer has to be processed for synesthesia to be elicited, and the relationship of synesthetic colors to the “real” colors triggered by wavelengths of light, with neuroimaging studies particularly focusing on the involvement of color-selective regions of the brain (V4). In the present disquisition, we develop the idea that synesthesia may represent an anomaly of *object knowledge*, akin to knowing a banana is yellow. Our starting point is the growing evidence that conceptual-level information is critical for synesthesia to arise and that synesthetic colors may more closely resemble color memory than color perception.

We first examine some influential claims that synesthetic colors are the same as the color experiences induced by wavelengths of light (henceforth “real” or “actual” colors). These studies are often interpreted as demonstrating that synesthesia is a “genuinely perceptual” phenomenon. We clarify the relationship between these behavioral data and the dominant neurocognitive models in the field, with a scrutiny on some crucial aspects of the data that are not taken into account in the major models. We then review recent behavioral and neuroimaging studies that suggest conceptual knowledge is pivotal in our understanding of synesthesia. In particular, these data demonstrate that synesthesia and our general conceptual knowledge of object attributes show similar psychophysical characteristics and may recruit common neural mechanisms. Finally, building upon the literature of “ideasthesia” (i.e., synesthetic experience with its roots in ideas or concepts; see Jürgens and Nikolić, 2012) we propose a conceptualization of synesthesia as a benign anomaly of the general mechanisms for representing object concepts. Incorporating the current understanding of semantic memory and its neural substrates, this framework explains many contemporary findings in the field and provides testable hypotheses for future work.

## PART 1: CONTROVERSIES OVER THE MECHANISMS UNDERLYING SYNESTHESIA

There are two dominant proposals regarding the neurophysiological basis of synesthesia. The *disinhibited feedback* theory suggests that synesthesia results from a “glitch” of the inhibitory circuitry that fails to suppress crosstalk between brain areas, which is normally inhibited in the non-synesthetic brain. According to different versions of this view, the disinhibition may occur in the feedback from multi-modal regions (e.g., superior temporal sulcus; Grossenbacher and Lovelace, 2001) or from areas involved in executive control (e.g., prefrontal cortex; Cohen Kadosh et al., 2009) to unimodal sensory areas. Some early evidence showing synesthesia-like experience elicited by hallucinogens has been interpreted in favor of this hypothesis such that the drug stifles normal inhibitory neural mechanisms (Grossenbacher and Lovelace, 2001). More recently, Cohen Kadosh et al. (2009) used hypnosis to elicit synesthesia-like experiences in individuals without synesthesia. They hypnotized four non-synesthetes and gave them associations between colors and numbers. These participants were then given post-hypnotic suggestions and performed a visual detection task designed by Smilek et al. (2001). Results showed that hypnotically induced “colors” affected detection of digits. Cohen Kadosh et al. (2009) argue that hypnosis mitigates inhibitory mechanisms, hence allowing a temporary emergence of synesthesia. This intriguing result needs to be considered with a few caveats. First, although there is evidence showing that hypnosis leads to a reduction in regional activation levels of the anterior cingulate cortex (Rainville et al., 2002), it is still unclear how such modulation occurs at the neuronal “excitation vs. inhibition” level. By the same token, the behavioral effect of hypnosis, albeit curious, does not clearly demonstrate alteration in inhibitory circuitries. Second, the induced effect in the hypnotized participants was much larger than that reported by Smilek et al. (2001) in a single synesthete (discussed in more detail in the later Section “Does Synesthesia Depend on Identification of Inducing Stimuli?”), raising the concern that the effects may not be analogous. At this point, therefore, the disinhibited feedback theory remains a plausible mechanism for synesthesia yet lacks direct evidence.

The other dominant view, the *cross-activation* theory, suggests that excessive neural connections between cortical areas underlie synesthetic experiences. In the seminal version of this theory, Ramachandran and Hubbard (2001b) proposed that synesthesia arises from direct cross-wiring of neural tracts between the adjoining areas processing inducers and concurrents. Grapheme–color synesthesia, then, would occur due to the grapheme area directly triggering activation of color area V4 via anomalous connectivity. A revision of this original hypothesis, which acknowledges the contribution of attention in binding color and visual word form, is the two-stage cascaded cross-tuning model (Hubbard, 2007; Brang et al., 2010; Hubbard et al., 2011). The major change is that although the anomalous color is initially generated via cross-activation between the grapheme area and V4, this “feature” still needs subsequent binding to create a unified percept. This binding is proposed to occur at a later stage, mediated by attentional mechanisms of the parietal lobe. This two-stage processing was proposed primarily to explain the mechanisms of grapheme–color synesthesia, but has recently been extended to account for other forms of synesthesia (Hubbard et al., 2011). A similar proposal to the cross-activation model is the re-entrant processing model (Smilek et al., 2001). In addition to “cross-talk” between relevant brain areas (e.g., areas processing form and color), this proposal includes feedback from higher-level areas (such as the anterior inferior temporal lobe) that represent the concept or meaning of the inducer (Smilek et al., 2001; Hubbard et al., 2011). This version of the cross-activation hypothesis has received less attention to date, but serves as a starting point for our discussion of the potential for higher-level areas to link inducers and concurrents.

The emphasis in the two dominant theories has very much been on the perceptual side of the system. This is also reflected in the primary focus of many neuroimaging studies of synesthesia: many studies have been designed to test whether synesthetic color is handled by a color-selective section of the fusiform gyrus (i.e., V4), with inconsistent results. Some studies find that synesthetic color correlates with V4 activation in some synesthetes and interpret this as synesthetic experiences resembling actual perceptual events (e.g., Hubbard et al., 2005; Sperling et al., 2006), whereas other studies fail to find a V4 response to synesthetic color (e.g., Rich et al., 2006; Hupé etal., 2012; Sinke et al., 2012b). There is also inconsistency between behavioral studies, with some claiming that synesthetic color behaves just like real color (e.g., Ramachandran and Hubbard, 2001a; Kim et al., 2006) and others discovering clear differences between the two types of colors (e.g., Edquist et al., 2006; Hong and Blake, 2008; Arnold et al., 2012). We review the relevant behavioral and neuroimaging studies that contribute to this debate in the following sections.

Most of the evidence reviewed here is based on grapheme–color synesthesia, due to it being the most widely-studied form (Rich et al., 2005; Simner et al., 2006; Novich et al., 2011). We do not, as yet, have a good basis for determining the likelihood that different forms of synesthesia differ fundamentally in their underlying mechanisms, but here we attempt to conceptualize a general form of the phenomena.

### DOES SYNESTHESIA DEPEND ON IDENTIFICATION OF INDUCING STIMULI?

One of the key issues in gauging the role conceptual processing has in synesthesia is to determine how “deeply” an inducer needs to be processed for synesthesia to arise. If synesthesia is triggered by “low-level” physical features (e.g., Ramachandran and Hubbard, 2001b), changing the appearance of an inducer should result in alteration of synesthetic percepts. If, in contrast, it is the meaning or the concept that elicits synesthesia, any stimulus that is identified as a particular inducer (e.g., A, a, and 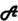 index the same concept) should elicit the same color. Subjective reports are mixed on which of these descriptions is accurate.

Rich et al. (2005) reported, based on questionnaire responses from 150 grapheme–color synesthetes, that the anomalous color experiences typically occur regardless of the modality of the inducer (seen, heard, or even just thought about). Moreover, changes in the appearance of an inducer (font, size, etc.) were only occasionally reported to impact on synesthetic color, suggesting it may be the lexical representation triggering color experiences rather than the physical appearance. In addition, the semantic context of an item has a strong effect on the perceived color: an identical stimulus is reported to elicit different colors depending whether it is interpreted as a digit or a letter under different contexts (e.g., 8**º** vs. g**º** Myles et al., 2003; also see Dixon et al., 2006). By contrast, other synesthetes have reported subtle differences in the saturation of color for different fonts or cases (e.g., E vs. ε or A vs. a; see Witthoft and Winawer, 2006) or only have synesthesia for visually presented but not spoken letters (Arnold et al., 2012). In our large-scale surveys, however, this occurs for a very small proportion of synesthetes, as is also reported by Simner (2012). In general, for the majority of synesthetes, it is the concept of a letter that determines the color rather than its low-level visual characteristics.

Dixon et al. (2000) objectively verified that synesthesia could be elicited by the concept of an inducer. They presented a digit (e.g., 4), an operator (e.g., +), a second digit (e.g., 3), and finally a color patch. They had a synesthete, “C,” name the color patch as quickly as possible, and then report the solution to the arithmetic problem. C showed a reliable congruency effect – her naming times were slower when the display color of the target patch was incongruent with the synesthetic color induced by the arithmetic solution, compared to when they were congruent. This indicates that synesthetic color can be triggered by a mental concept, without the inducing stimulus being physically present.

In a subsequent study by the same research team, Smilek et al. (2001) briefly presented a black digit on a colored background that either matched or mismatched C’s synesthetic color for the digit. C was poorer at identifying the digit when it was presented on a congruently colored background relative to an incongruent one, as if it was camouflaged by the background color. Some aspects of these data are puzzling. For example, if the digit could be effectively concealed by the synesthetic color matching the display background color, C should perform worse than non-synesthetic controls. However, C’s performance in the congruent condition was actually comparable to controls; her performance was just better than controls in the incongruent condition. Based on their data, Smilek et al. (2001) propose that synesthesia arises when the concept/meaning of an inducer is activated but does *not* require conscious identification.

In contrast to the results of Smilek et al. (2001), Mattingley et al. (2001) found that synesthesia depended critically on *conscious* identification of the inducer. They presented 15 synesthetes with a variant of a synesthetic congruency task (Wollen and Ruggiero, 1983) in which the synesthetic color elicited by a letter inducer prime was either congruent or incongruent with a succeeding target color to be named. They showed a robust synesthetic priming effect from the letter prime on subsequent color naming times when the prime was clearly visible. This priming effect disappeared when the inducer prime was rendered invisible by a backward mask. The unseen primes still elicited a priming effect on a letter naming task, indicating some unconscious processing of the letter, at least to the level of orthographic representation. Mattingley et al. (2001) interpreted these results as synesthetic color only arising when the inducer is consciously identified. Later findings have been consistent with this claim, demonstrating that synesthetic congruency effects can be abolished when attentional resources are not sufficient for conscious report of a letter, such as in the middle of the attentional blink (Rich and Mattingley, 2010). Concurrently, other studies have demonstrated that reducing attentional resources available for processing the inducer (presumably hampering identification) attenuates the otherwise reliable impact of synesthetic color on color naming (Rich and Mattingley, 2003; Mattingley et al., 2006; Sagiv et al., 2006).

The studies reviewed above suggest that access to the conceptual representation of an inducer plays a critical role in eliciting synesthesia. However, the extent to which meaning without full conscious identification can elicit synesthesia is unclear. Smilek et al. (2001) emphasize that access to the concept/meaning of the inducer should be sufficient to trigger synesthetic colors. Access to lexical or pictorial meaning can occur prior to identification, leading to masked priming effects (e.g., Dell’Acqua and Grainger, 1999). In synesthesia, however, the masked priming data (Mattingley et al., 2001) suggest that implicit processing without conscious identification is not enough to elicit synesthesia (at least, not sufficient to cause a reliably detectable synesthetic congruency effect). Thus, it seems clear that access to a concept is important, and that the depth of processing needs to be considerable (probably to full conscious identification) before synesthesia arises.

### IS SYNESTHETIC COLOR EQUIVALENT TO REAL COLOR?

Another major focus of synesthesia research has been whether induced synesthetic color is equivalent to “real” color. Subjective reports again provide mixed information about this. On the one hand, synesthetes sometimes report that their synesthetic colors can be as conspicuous as real colors (Ramachandran and Hubbard, 2001a, 2001b), and on the other hand, they can clearly distinguish synesthetic colors from the actual colors of triggering graphemes. Despite some influential claims in early studies, objective data are generally consistent in demonstrating a difference between synesthetic and real colors.

In an influential study, Ramachandran and Hubbard (2001a) demonstrated that synesthetic color facilitated visual search – a unique synesthetic color caused an obscure shape to “pop out” from surrounding items of a visual display. It is well-established that actual color causes “pop-out” during visual search: when a target has a unique color, the number of distractors has minimal effect on the reaction time (RT) to detect the target (Treisman and Gelade, 1980). This efficient search usually leads to a shallow or flat search slope of the RT by set-size function, demonstrating that color is registered rapidly into its sensory channels and can be effectively used to guide attention. In their well-known synesthetic “pop-out” study, Ramachandran and Hubbard (2001a) had two synesthetes search through an array of black digits for an embedded contour composed of identical digits. Compared to non-synesthetic controls, the synesthetes were more accurate in identifying the embedded target (controls: 59.40%; synesthetes: 81.25%; chance level was 25%). In a later paper (Ramachandran and Hubbard, 2001b), the authors interpreted this result as synesthetic color “popping out” like real color and facilitating visual search.

A synesthetic pop-out effect would be provocative, as it conflicts with what is known about the role of attention in letter recognition (e.g., Mackeben, 1999), and indeed, with our claim that synesthetic color arises *after* attentional processing of the inducer (e.g., Mattingley et al., 2006; Rich and Mattingley, 2010). Subsequent visual search studies, however, found that although synesthetic color occasionally bestows an advantage during visual search, it does *not* lead to the pop-out effect. Palmeri et al. (2002) found a synesthete outperformed controls during a traditional visual search task with a single target among multiple homogeneous distractors. However, the slope of the RT by set-size function was much steeper than the typical flat slope of the pop-out effect. Moreover, the effect only occurred when the *distractors* elicited synesthesia, making it clear the effect was *not* related to the synesthetic color of the target. Edquist et al. (2006) and Sagiv et al. (2006) also had synesthetes perform visual search tasks. In neither study (sample size: 14 synesthetes by Edquist et al., 2006 and 2 synesthetes by Sagiv et al., 2006) was there any evidence of a synesthetic advantage over non-synesthetic controls. Whereas a unique chromatic letter was immediately manifest, an achromatic letter could only be found via an effortful search that did not differ from controls, indicating *no* synesthetic pop-out. Laeng et al. (2004) also tested a synesthete using a visual search task. Initially, these authors found that synesthesia could lead to an advantage, but only when the target was within the central focus of attention. A later study by Laeng (2009) suggested that pop-out could occur if the target and distractor letters were carefully chosen to elicit colors far distant in color space. This remains the only case to date that resembles a truly efficient search function.

Returning to the embedded figures task, Ward et al. (2010) tested 36 synesthetes and 36 controls and recorded synesthetes’ trial-by-trial descriptions of their synesthetic experiences. They observed a general superiority of synesthetes in accuracy (synesthetes: 41.4%; controls: 31.5%), replicating the original Ramachandran and Hubbard (2001a) case-study results, albeit with a smaller (~10%) advantage for synesthetes. However, most of their synesthetes reported *no* color experience during search, and synesthetic color only seemed to emerge when a letter was attended. They concluded this was inconsistent with “pop-out,” based on the subjective reports and the fact that the accuracy advantage for synesthetes was far less than one would expect from actually colored stimuli. Rich and Karstoft (2013) modified the embedded figure task to manipulate set-size, allowing a direct objective test of the pop-out hypothesis. With eight synesthetes and eight matched controls, they replicated the advantage for synesthetes over controls in accuracy at the largest set-size (64 items; synesthetes: 1.87% errors; controls: 11.56% errors) but found no differences in RTs/search slopes that would be indicative of a synesthetic “pop-out” effect. At this point, despite subtle advantages in some visual search studies for some synesthetes, there is little evidence that synesthetic color pops out like real color.

There are alternative explanations to explain the occasional advantage of synesthetes in visual search-type tasks. For example, Mattingley (2009) raised a possibility that synesthetic color may facilitate the grouping of items and confine search scope (also see Ward et al., 2010; Rich and Karstoft, 2013 for discussion of the grouping account). In addition, a recent study (Rappaport et al., 2013) reported that coloring an object with its familiar color (e.g., yellow corn) led to more efficient visual search (slope: 10 ms/item), compared to it being in an atypical color (22 ms/item). Note that the facilitation by color familiarity/memory is still inferior to pop-out searches for real color, which often gives slopes close to 0 (Wolfe and Horowitz, 2004). Such findings resemble the pattern seen in synesthetic color improving visual search, implying that the facilitation originates from well-learnt conjunctive representations, be it synesthetic or ordinary mnemonic, reducing attentional constraints but causing no pop-out. Furthermore, as we will discuss later, the resemblance raises the hypothesis that the human brain employs the same mechanisms to represent synesthetic color and the knowledge about canonical object color.

Other studies using psychophysical measures of low-level visual properties also suggest that synesthetic color is distinct from real color. For example, synesthetic color does *not* induce chromatic adaptation (Hong and Blake, 2008), the simultaneous color contrast effect (Nijboer et al., 2011a), or the color constancy effect (Erskine et al., 2012). Although there are some claims that synesthetic color behaves like actual color to affect bistable apparent motion (Kim et al., 2006) and to induce the watercolor illusion (Kim and Blake, 2005), these studies tested only two synesthetes and used designs that are not criterion-free. For instance, Kim et al. (2006) asked the synesthetes to decide whether two alternating visual arrays of letters appeared to move leftward or rightward and found that letters that induced the same color tended to be “grouped” together, which biased motion trajectory of the arrays. However, the need to select between two alternatives could result in an effect that simply reflects the tendency to use color similarity to guide a decision. With this design, there is no way of telling whether synesthetic color truly biases perception or simply the decisional criterion. Overall, there are clear differences between synesthetic color and real color, suggesting there must be crucial differences in the mechanisms that underpin the two forms of color experiences.

### CONTROVERSIES OVER THE CATEGORIZATION OF SYNESTHETES

One explanation for the discrepant results regarding how closely synesthetic color resembles real color is that different mechanisms underpin synesthesia for different sub-groups, and, for at least some synesthetes, their evoked colors *are* akin to “real” colors. Dixon et al. (2004) distinguish “projectors,” who describe synesthetic color as appearing “out in space,” from “associators,” who describe the color as “in the mind’s eye.” They suggest that, compared to associators, the synesthetic color of projectors emerges earlier in the visual system. In another proposal, Ramachandran and Hubbard (2001b) differentiate between “lower synesthetes,” for whom synesthesia is induced by sensory stimulation, and “higher synesthetes,” for whom it is caused by the meaning of the inducer. In lower synesthetes, the cross-wiring between the grapheme area and the color center (V4) is assumed to generate their synesthetic color whereas, in higher synesthetes, the wiring to the parietal lobe produces the anomalous color. These categorizations were originally proposed to reconcile contradictory results. However, they have now generated more debate.

Dixon et al. (2004) tested whether they could objectively distinguish “projectors” from “associators” using two versions of the synesthetic congruency task – naming the physical color of a letter or naming its synesthetic color. The display color and the synesthetic color on a given trial could either match (congruent) or conflict (incongruent). Their synesthetes were first separated by subjective report (seven associators and five projectors). Results showed that those classified as projectors had a larger congruency effect (incongruent minus congruent RT) when naming displayed color than synesthetic color, whereas associators showed the opposite pattern (see Ward et al., 2007 for similar demonstrations with seven associators and seven projectors). However, other studies have failed to find differences between subjectively classified projectors and associators on visual search tasks. For example, Edquist et al. (2006) tested 14 synesthetes and found that their anomalous colors did not affect performance on a visual search task, regardless of whether the synesthete identified his/herself as an associator or a projector (based on initial responses to just the question of whether the color appeared “out in space” vs. “in my mind’s eye,” there were five projectors, seven associators, and two subjects who reported that neither of these fitted their experiences; also see below for the variation of reports across time). Sagiv et al. (2006) also tested two self-reported projectors on a visual search task and found no advantage from synesthetic colors. In a more recent study with a large sample size (27 associators and 9 projectors, classified based on responses to questionnaires), Ward et al. (2010) also failed to find any difference between the two types of synesthetes on a visual search task. As a final example, Nijboer et al. (2011b; also see Nijboer and Van der Stigchel, 2009) asked nine synesthetes (six associators and three projectors) to execute an eye movement to a target digit during visual search. The results showed that a target that usually elicited a synesthetic color did not lead to the pop-out effect (indexed by faster saccadic onset) in either the associators or the projectors. One further issue with this dichotomy is that the subjective reports of “in the mind’s eye” vs. “out in space” may be unreliable, switching from one description to the other between two points in time (Edquist et al., 2006). This variability presumably reflects the difficulty in using language to describe the subjective experience. Thus, although it is always possible that for some synesthetic individuals synesthetic colors do behave like real colors, there is little evidence of reliable behavioral differences at this point.

Some neuroimaging studies have claimed to lend support to the “associator vs. projector” distinction. Rouw and Scholte (2010) used voxel-based morphometry (VBM) to compare the volume and density of neuronal gray matter (GM) between 16 projectors and 26 associators, compared with 42 non-synesthetic controls. They also used functional magnetic resonance imaging (fMRI) to look at the functional response to graphemes (contrasting color-inducing graphemes vs. non-inducing symbols), relative to 19 non-synesthetic controls. Synesthetes were classified as projectors and associators based on their scores on a questionnaire probing the subjective locus of their synesthetic colors. In structural measures, projectors had increased GM in the cortical areas for perception and action (i.e., the visual cortex and other sensorimotor areas), whereas associators had more GM in the regions involved in memory encoding and retrieval (i.e., the hippocampus and parahippocampal gyri). Their fMRI data also revealed that, compared to projectors, associators showed greater activity in the bilateral parahippocampal gyri, which mapped to the same regions that showed structural alternation in the VBM analyses. The authors suggest that such neuroanatomical differences drive the individual variability in the subjective locus of synesthetic color (internal “in the mind’s eye” vs. external “out in space”). Although this inference is intriguing, others have raised concerns about the stringency of the analyses. Hupé et al. (2012) note that it is not clear whether Rouw and Scholte (2007), 2010 applied correction for multiple comparisons to data over the whole brain and suggest that not including brain size as a covariate can be problematic. In addition to the question about whether the analyses have enough rigor, there is conflicting evidence regarding the “distribution” of the two hypothesized forms of synesthetes: in an earlier study by the same authors, Rouw and Scholte (2007) tested 18 synesthetes and found their scores on the “associator vs. projector” dimension appeared more evenly distributed along the spectrum and formed a positive correlation with the fractional anisotropy (an index of fiber density or directional coherence in white matter tracts) of the right temporal cortex, implying the variability is continuous (also see Eagleman, 2012). In their later study (Rouw and Scholte, 2010), which included these 18 synesthetes in a final sample of 42, the distribution of self-reports looked more bi-modal rather than falling along a continuum. The Rouw and Scholte (2010) paper remains the most comprehensive imaging paper in synesthesia research to date. Other neuroimaging studies using fMRI (van Leeuwen et al., 2010)^1^ and electroencephalography (EEG; Gebuis et al., 2009) have failed to find reliable differences between associators and projectors in regional haemodynamic responses and electrophysiological waveforms. Overall, we tend to agree with Eagleman (2012) that it is difficult to know whether we are looking at the extremes of a continuum or a categorical distinction between two groups differing fundamentally in the spatial frame of their synesthetic colors.

Compared to the “projector vs. association” distinction, there is less empirical data on the “lower vs. higher” categorization of synesthetes. Hubbard et al. (2005) emphasized the potential importance of individual differences in their fMRI study. They tested six synesthetes using their embedded figures task (discussed previously) and a crowding task. In the latter task, participants had to identify a target letter flanked by distractors in the visual periphery, with all stimuli being achromatic. Identifying the target was difficult as distractors “crowded” the target, rendering it unrecognizable. In the embedded figures task, five of the six synesthetes performed better than their controls in their accuracy of identifying the embedded shape. For the crowding task, three of the six synesthetes had a slight advantage over their controls in correctly identifying targets, which the authors interpreted as synesthetic color mitigating visual crowding (note that no difference was found at the group level). The authors then examined the response of visual cortical regions in synesthetes and controls presented with achromatic graphemes. They reported significantly higher V4 activation in synesthetes than controls (albeit with a liberal statistical approach, a one-tailed test that assumes *a priori* higher V4 activity in synesthetes than controls; see Hupé et al. 2012). Further, the authors found a significant correlation between performance on the crowding task and the strength of V4 activity such that synesthetes with greater V4 activation tended to perform better on the visual crowding task. The authors propose that individual differences in the ability to perform the crowding task map onto the “lower” (better performance, more V4 activity) vs. “higher” (average performance, less V4 activity) synesthete distinction^2^. As they note, however, it is not possible with such a small sample size to distinguish sub-groups from polar extremes of a continuum.

There is a general consensus among researchers that the salience or intensity of synesthetic experience varies between individuals. The challenge lies in finding whether this variation truly indicates fundamental differences, perhaps qualitatively involving distinct neural mechanisms, such that we should categorize synesthetes into sub-groups, or whether it reflects individual variability within the same basic mechanism.

### DEBATES CONCERNING THE NEURAL BASIS OF SYNESTHESIA

The notion that there must be a difference in neuronal “wiring” between synesthetes and non-synesthetes has been popular among scientists and the general public alike. As discussed earlier, the *disinhibited feedback* theory suggests that synesthesia results from a deficiency of inter-neuronal inhibition. The evidence for this theory (e.g., synesthesia-like experience elicited by drug or hypnosis) is all behavioral and does not provide direct support for the existence of malfunctioning neuronal inhibition. Further, a recent paper systematically compared developmental synesthesia to drug-induced similar experiences and found that the two have more differences than commonalities (Sinke et al., 2012a). Neuroimaging studies have provided more direct examination of the predictions of the *cross-activation* theory. In the case of grapheme–color synesthesia, the cross-activation view assumes that synesthetic color is first induced by the grapheme area directly triggering activation in V4, the specialized area for processing color; at a subsequent stage, the attentional mechanisms mediated by the parietal lobe act as “glue” to combine synesthetic color with visual word form. Although the involvement of the parietal lobe in synesthesia is generally agreed upon amongst researchers, there is debate about the first hypothesized stage. There are two principle points of contention.

The first issue with the cross-activation theory is that there is still debate about whether the human brain really hosts a cortical region specialized for processing visual word form. Reading text activates the left lateral occipitotemporal sulcus/the left fusiform gyrus, a site often referred to as the visual word form area (VWFA; see Cohen et al., 2002; McCandliss et al., 2003). This site is reliably activated by graphemic stimuli, regardless of different scripts or languages. Moreover, brain lesions of the VWFA cause pure alexia, a selective deficit in word recognition (Cohen et al., 2000). Such evidence has led to the theory that this left ventral occipitotemporal section of the brain is specialized for conjoining graphemic features into a word shape, prior to the stimulus accessing its phonological and semantic codes (Dehaene and Cohen, 2011). However, the specificity of this area to graphemic processing has been challenged. For instance, Price and Devlin (2003) found that this “visual word form” area could also be activated by images of animals and vehicles. Devlin et al. (2006) also found that this area responded to the semantic relations between words, suggesting that it does not exclusively represent visual word form. Two recent studies explored the functionally connectivity of the VWFA (Vogel et al., 2012a) and contrasted its response to words and line drawings (Vogel et al., 2012b). Results showed that the activity of this region did not functionally co-vary with other reading-related regions and showed no preferential response to words, which the authors interpret as evidence that it is a more general visual processor used in reading but also in other visual tasks. Given the debate, it may be premature to conclude the putative contribution of this region to synesthesia is, as the cross-activation view assumes, the visual analysis of graphemic components. It is possible it reflects non-perceptual processing, such as the semantic representation of a stimulus.

The second, perhaps more problematic, issue for the cross-activation account is whether synesthetic color recruits V4. Some studies have observed V4 activation when synesthetes view or hear graphemes (Nunn et al., 2002; Hubbard et al., 2005; Sperling et al., 2006). However, other studies did not find V4 activation during synesthesia (Rich et al., 2006; Hupé et al. 2012; Sinke et al., 2012b). Hupé et al. (2012) conducted a thorough review of the literature to assess the origin of these discrepancies; they claim that all imaging studies in synesthesia that report V4 activation in synesthesia use overly liberal statistical criteria. In the study by Nunn et al. (2002), the areas activated by synesthetic color did *not* actually overlap with those by real color. In the study of Sperling et al. (2006), there was no correction for multiple comparisons for statistical tests; despite this liberal criterion, they found V4 activity in only two out of four synesthetes. Finally, in Hubbard et al. (2005), the criterion was also liberal (one-tailed tests assuming higher V4 activation in synesthetes than in controls). Of course, these statistical issues are not limited to studies reporting V4 activation, but the Hupé et al. (2012) critique emphasizes the potential for such analysis decisions to lead to a misperception of the robustness of such findings. It is therefore by no means unequivocal from the data presented to date that synesthetic color activates V4.

In addition to the issue of overly liberal statistical criterion, Mattingley (2009) speculates that the greater V4 activation in synesthetes in some studies may be confounded by mental imagery. Various lines of inquiry seem to give credence to this suggestion. Barnett and Newell (2008) reported that synesthetes (*n* = 38) scored higher than controls on the Vividness of Visual Imagery Questionnaire (VVIQ), a test probing the vividness of their visual imagery. Although this merely suggests that synesthetes may have more vivid imagery and an inclination to imagine, there are two pieces of evidence linking this behavioral result to neural processing. First, Cui et al. (2007) showed that individuals (*n* = 8) who score higher on VVIQ tend to show higher activity in the early visual cortex (Brodmann’s areas 17 and 18, regions of interest (ROIs) defined using a neuroanatomical database) when performing mental imagery. Second, Rich et al. (2006) used a color perception localizer (colored minus gray-scale Mondrians) to define ROIs in the visual cortex responsive to colored stimuli in seven synesthetes and matched controls. Within these color-responsive ROIs (voxels passing small volume correction), they found that voluntary color imagery resulted in activation for both synesthetes and controls in the fusiform gyrus, a location consistent with the literature definition of V4. These findings together imply that V4 activation in synesthesia studies could potentially reflect synesthetes imagining color, rather than synesthetic color *per se*. A recent study lent further support to this imagery explanation of V4 activity: Sinke et al. (2012b) found that when synesthetes and controls were matched for their scores on VVIQ, there was no difference in V4 activation between the two groups. That is not to say that synesthesia is only voluntary imagery, but just that in a scanning environment where synesthesia is the focus, participants who are trying hard might utilize color imagery, which consequently activates V4. We need more evidence before concluding that synesthesia originates from the cross-wiring between V4 and grapheme area, and that synesthesia and “real” color share neural bases.

Thus far, the data we have reviewed shows a discrepancy between behavioral data and the assumptions made by the dominant theories about the neural mechanisms of synesthesia. We argue that behavioral evidence suggests that synesthesia is strongly reliant upon and influenced by the *conceptual* representation of inducers and has attributes that are distinct from those of actual color. The neurocognitive theories of synesthesia (especially the cross-activation view) focus on the grapheme area and V4, postulating that synesthesia depends on the feature-level (lines, intersections, etc.) perceptual processing of a grapheme and resembles real color. We need a framework for synesthesia that is better able to integrate a broader gamut of behavioral and neuroimaging findings. In the following section, we expand upon the role conceptual knowledge has in synesthesia, and propose a conceptual-mediation neurocognitive framework.

## PART 2: THE ROLE OF CONCEPTUAL KNOWLEDGE IN SYNESTHESIA

As reviewed in Part 1, there is good evidence that substantial processing of an inducing stimulus, including access to meaning/concept, is necessary to elicit synesthesia (e.g., Dixon et al., 2000; Mattingley et al., 2001; Rich et al., 2005; Nijboer and Van der Stigchel, 2009; Rich and Mattingley, 2010; Nijboer et al., 2011b; Simner, 2012). Research into the nature and effects of synesthetic experience has also highlighted distinctions between synesthetic and real colors in terms of their psychophysical characteristics (e.g., Edquist et al., 2006; Sagiv et al., 2006; Hong and Blake, 2008; Arnold et al., 2012; Erskine et al., 2012). The overall pattern of contemporary findings suggests a need for a framework that emphasizes the role of high-level processing in synesthesia, such as conceptual knowledge.

**FIGURE 1:**
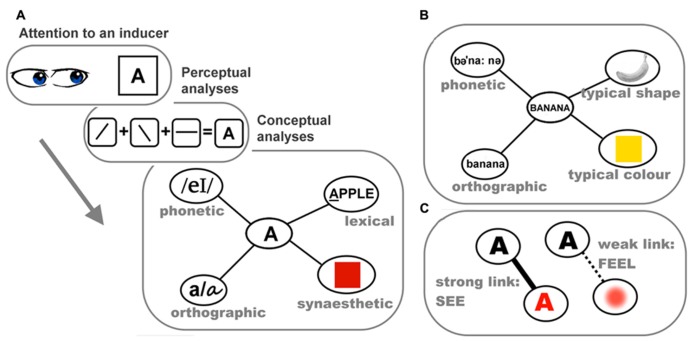
**The conceptual-mediation model of synesthesia.** **(A)** Schematic of the processes involved in the elicitation of lexically induced synesthetic color experience. In this information processing stream, attention to the inducer letter “A” serves as the gatekeeper for subsequent analyses. The perceptual system then analyses how lines are conjoined, deriving the visual word form of “A.” The visual representation of “A” subsequently activates the core lexical concept of “A,” which in turn excites various associated nodes of conceptual properties in a semantic network, including its red synesthetic color; **(B)** a schematic illustration of the semantic network of the concept of banana and its related conceptual features; **(C)** the salience/vividness of synesthetic color may depend on the strength or directness of the link between the lexical presentation of “A” and its synesthetically red color.

**Figure 1A** shows a schematic illustration of our proposed stages involved in the elicitation of synesthetic color: initially attentive processing of an inducer leads to perceptual analyses of its physical attributes (e.g., lines, curves, conjunctions, etc.). A series of perceptual processes culminates in the identification of that stimulus at a conceptual level, regardless of the modality of presentation. The action of identification may differ between stimulus types (e.g., letter identification is individuating the lexical identity, whereas for a sound there can be the awareness that a sound has occurred, recognition of instrument or speaker, through to individuation of that sound with a label, such as *middle C* or *F-sharp minor*). To this point, processing in synesthetes and non-synesthetes should be identical. Activation of the stimulus concept spreads through a semantic network: the activation propagates from the core conceptual representation of the inducer to its associated nodes of conceptual attributes, which for synesthetes, includes a link to the concept of a color absent (or weak) in non-synesthetes. We conceptualize this as a similar linkage to the way in which the concept of a banana includes its canonical color (**Figure 1B**). The end-point of this cascade of cognitive processing is an experience of synesthetic color, with variability in the individual experience from a “*feeling*” of color to a strong “*percept*” of color, perhaps depending on the strength and distance of the connections within the neural network. A strong link bridging the ensemble of neurons coding lexical meaning in the semantic network to those coding color in the perceptual areas might travel through the occipitotemporal regions involved in color-related processes representing *memorized* and/or *perceived* color, resulting in a vivid percept of color. By contrast, a weaker link connecting lexical and color representation within the semantic network, or one that persists only partway down the system toward the perceptual areas, may only give a faint percept or a “feeling” of color without clear visual components (**Figure 1C**).

How can this conceptual-mediation model account for the range of behavioral and neural data? First, the role of attention is clear. Without attention to an inducer, there is no identification, and therefore the process does not kick off. Second, the abstract identity of the inducer is linked to its synesthetic color at a conceptual level and the processing propagates back down the network toward more perceptual representations. This framework encompasses the top-down effects including semantic context, as well as allowing for the individual variability in the “percept-like-ness” of the experience. Finally, as elaborated later, when non-synesthetes are asked to make voluntary associations between (for example) sounds and colors, there are systematic patterns that are strikingly similar to those seen in the inducer-concurrent pairings of synesthetes. There is evidence that these trends of cross-modal mappings observed in non-synesthete are mediated by semantic knowledge, again suggesting it may be a groundwork subserving synesthetic and ordinary phenomena. We propose that synesthesia has cognitive roots in the semantic knowledge base that we all share, rather than requiring a fundamentally different neural network of connections.

One prediction of the conceptual-mediation model is that synesthetic colors should be more similar to high-level color knowledge than to perceiving “real” color, because the critical link occurs between concepts – the link is between, for instance, the *concepts* of lexical entity “A” and perceptual property “red.” There are a few recent studies suggesting that this may indeed be the case. Results of recent fMRI and brain stimulation studies also hint that synesthetic color and color knowledge may recruit common neural substrates, beyond the realm of V4. In the following sections, we review this recent evidence and further discuss the proposal that synesthesia relies on the cognitive and neural mechanisms of conceptual knowledge. We also outline how this conceptualization can better explain why non-synesthetes show strikingly similar trends to the unusual experiences of synesthetes when asked to associate graphemes/sounds with visual attributes, and the implicit cross-modal mappings we all share.

### GRAPHEME–COLOR SYNESTHESIA RESEMBLES COLOR KNOWLEDGE/MEMORY

The conceptual-mediation model predicts that synesthetic colors should more closely resemble high-level color knowledge, where color is an intrinsic attribute contributing to the concept of an object (e.g., knowing a banana is yellow) than low-level color perception (e.g., perceiving a shade of yellow hue). Nijboer et al. (2011a) tested whether synesthetic color could elicit the simultaneous color contrast effect, a phenomenon reflecting early visual processing. They presented an achromatic grapheme on a colored background and had 12 synesthetes adjust the color of a white hash sign to match their synesthetic color. Twelve controls performed the same task with physically colored graphemes (matched to their synesthetic counterparts). The key manipulation was that the background and grapheme could have colors similar in hue or opponent colors. Controls demonstrated the typical simultaneous color contrast effect (Valberg and Lange-Malecki, 1990): when the grapheme and background had opponent colors (e.g., a green background and a red grapheme), responses tended to “offset” the background color. Specifically, a green background made a red grapheme seem redder than it actually was (color contrast), so controls added more red than what was present in the reference stimulus (i.e., grapheme). Synesthetes showed an inverse pattern to that of controls such that their responses tended to “conform” to the background color. When a grapheme induced a *red* synesthetic color appearing on a *green* background (opponent colors), synesthetes added insufficient red to match their synesthetic color. When it was a *red* synesthetic color on a *red* background (similar colors), they added excessive red.

The absence of the typical color contrast effect from synesthetic colors concurs with earlier studies that early mechanisms do not contribute to synesthesia (e.g., Hong and Blake, 2008). However, it is the reverse pattern of synesthetic colors relative to the typical effect of real color that raises an interesting possibility: synesthetic color may be an anomalous case of *object color knowledge* (e.g., knowing that bananas are typically yellow). There is evidence that color knowledge can affect perceived color appearance (Hansen et al., 2006), and also that the representation of high-level color memory is less precise then real color (Knill and Richards, 1996). Thus, Nijboer et al. (2011a) suggest that, similar to color knowledge, the perceptual representation of synesthetic color may be less solid and tend to fluctuate more easily than real color. This difference in perceptual characteristics relative to real color may lead to synesthetic colors assimilating into the background, resulting in the reverse pattern. To explain the potentially pliable nature of synesthetic color, Nijboer et al. (2011a) drew an analogy to the impact of perceptual noise on shape perception: when the contour of a stimulus is obscured by noise and becomes less concrete, shape representation gradually assimilates into the clearly defined background (Van der Kooij and te Pas, 2009). Although it would be more cogent to have a direct comparison between synesthetic colors and general object knowledge with regard to the effect on perceived color appearance, these data support the notion that high-level color knowledge could form the basis for synesthetic colors.

The idea that synesthetic colors may resemble a higher-level color representation such as color knowledge or color memory has been directly addressed in another recent study. Arnold et al. (2012) tested six synesthetes who reported that seeing a printed grapheme evoked a color experience whereas hearing a spoken grapheme generated no color. The synesthetic color of a heard grapheme could nonetheless be retrieved from memory. Synesthetes adjusted a color patch to match their synesthetic colors. The graphemes were presented in two different modes (printed/spoken). Thus, measures of precision (distance in color coordinates between matched and synesthetic color) could be derived and compared between when it was *online* (seen concomitantly with a grapheme) and *offline* (recalled from memory when hearing a grapheme – although note this was based on subjective report; it would have been ideal to have an objective measure of the difference in synesthetic colors between the two conditions). They found the degree of precision was comparable between colors elicited by printed and spoken graphemes, suggesting that synesthetic color perceived in real-time is perceptually equivalent to the color recalled from memory. In a control experiment, the precision of actual color seen in real-time greatly exceeded that of color memory. These results indicate that visible real color has a higher perceptual precision than color memory, consistent with Knill and Richards (1996). In contrast, synesthetic color, be it seen online or recalled offline, is perceptually analogous to color memory.

In sum, although synesthetic color may be as vivid as real color at the subjective level, recent psychophysical data suggests that it is akin to reinstatement of perceptual memory. This is a significant step forward in determining the nature of synesthetic experience, and the level at which the critical representation might occur. Such effects are consistent with the proposal that the critical process linking inducers to concurrents is conceptual, rather than entirely perceptual, in nature.

### SYNESTHETES SHOW SIMILAR TRENDS TO NON-SYNESTHETIC INDIVIDUALS

The comparison between synesthetes and non-synesthetes in their ways of associating inducing stimuli with perceptual attributes offers further clues regarding the contribution of conceptual processing in mediating synesthesia. In the case of grapheme–color synesthesia, large-scale studies looking for patterns across synesthetes suggest that *lexical/semantic knowledge* has an important role in mediating the coupling between lexical stimuli and colors. Rich et al. (2005) found a striking consistency among 150 synesthetes in their synesthetic pairing between graphemes and colors. For half the letters of the alphabet, certain letter–color pairs were reported significantly more often than would be expected by chance (e.g., “R” elicited red for 36% of the sample, “Y” elicited yellow for 45%, “O” elicited white for 56%, “D” elicited brown for 47%). Interestingly, similar trends were apparent for a group of non-synesthetic controls who were asked to pair colors with letters and numbers (i.e., controls reported most of the same letter–color associations as synesthetes). Simner et al. (2005) also reported a noticeable similarity between synesthetes and controls in their grapheme–color associations (e.g., in both groups, “A” tended to be red, “B” tended to be blue). Both studies suggest that grapheme–color synesthesia, whilst only manifest in a small percentage of the population, stems from mechanisms common to non-synesthetes, such as similar semantic knowledge or learning experiences. In non-synesthetes, retrieving this type of weak conceptual link (e.g., associating “A” with red) is a volitional mental operation. In synesthetes, however, accessing the links from inducers to concurrents is typically involuntary, implying a strong link between the lexical identity and color as a related attribute of the concept.

In addition to the associations between letters and colors, similarities between synesthetes and non-synesthetes have also been found in other types of synesthesia. As with grapheme–color synesthesia, there are hints that the similarities reflect contributions of high-level conceptual knowledge rather than low-level sensory processing. One example is auditory–visual synesthesia, a phenomenon where auditory stimuli elicit visual experiences.

**FIGURE 2:**
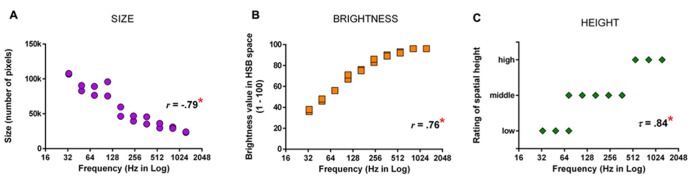
**Chiou et al. (2012) had synesthetes draw their synesthetic percepts in response to instrument notes using graphics software and analyzed how synesthetic visual features varied with auditory pitch.** **(A)** The relationship between pitch and the size of synesthetic objects as measured by the number of pixels in the drawings. **(B)** The relationship between pitch and the brightness of synesthetic color, indicated by brightness value (1–100). **(C)** The relationship between pitch and the location of synesthetic objects, indicated by categorical codings of synesthetes’ description. Asterisks indicate a statistically significant correlation (*p* < 0.05). Note that there are 20 data-points (2 repetitions by 10 notes) in each figure, but some data-points are concealed due to auditory pitch (Hz) being compressed in logarithmic scale. Redrawn based on **Figure 4** of Chiou et al. (2012) with permission of the publisher.

There is good evidence showing systematic trends in the way synesthetic visual experiences vary with acoustic features of sounds (e.g., pitch). These patterns are also present in the implicit mappings seen in non-synesthetes. Ward et al. (2006) had 10 auditory–visual synesthetes select their synesthetic colors in response to instrument sounds. Ten controls were asked to associate colors with the same sounds. Although the color chosen by synesthetes was more consistent than controls across two repetitions, there was a pattern in both groups: as auditory pitch got higher, the selected colors gradually became brighter, akin to the typical pitch–brightness mapping (Marks, 1975). This systematicity is not limited to the color dimension. We tested seven synesthetes for whom sounds induced synesthetic visual experiences and, in addition to asking about synesthetic colors, we documented other non-color features, such as size, shape, and location of the experience (Chiou et al., 2012). Their synesthetic experiences in response to instrument notes were usually colored geometric objects, often with a specific spatial location (in the mind’s eye). As illustrated in **Figures 2A–C**, there were systematic patterns such that as the auditory pitch got higher, the appearance of synesthetic objects became brighter in color, smaller in size, and higher in spatial location. This trend closely resembles the correspondences between auditory pitch and visual features documented in non-synesthetes (Spence, 2011), which we suggest implies a common mechanism underlying the two phenomena. We further speculate that such shared mechanisms operate at the stage of conceptual processing: there is accumulating evidence that cross-modal correspondences rely more on cognitive processing at an abstract conceptual level and perhaps less on mechanisms of the perceptual system. For instance, the mapping between pitch and location has been demonstrated to rely on recognition of a sound’s relative pitch height within a given context rather than acoustic feature of absolute pitch height (Chiou and Rich, 2012); the mapping between pitch and size has been found to be semantically mediated (Gallace and Spence, 2006); and the same set of conceptual regularities seem to guide whether various sensory attributes form a harmonious combination or not (Walker, 2012; Walker and Walker, 2012; Walker et al., 2012). Furthermore, the impact of some cross-modal mappings (e.g., pitch-size; loudness–brightness) on perceptual judgment has been shown to reflect biases at a post-perceptual decisional level, rather than perceptual-level phenomena (Marks et al., 2003; Keetels and Vroomen, 2011). Although whether different types of cross-modal mappings differ in their extent of reliance on “perceptual vs. conceptual” factors awaits more exploration (for discussion, see Spence, 2011), the current pattern of results seems to suggest a key role of concepts in various types of cross-modality correspondences. If synesthesia builds on these cross-modality mechanisms we all share (as argued by different research teams; see Ward et al., 2006; Chiou et al., 2012; Bankieris and Simner, 2013), it should similarly depend on conceptual processing.

There are also commonalities in the synesthetic mapping between brightness and magnitude. Cohen Kadosh et al. (2007) tested individuals with digit-color synesthesia and found the brightness value of synesthetic color was negatively correlated with numerical size. This resembles the patterns seen in non-synesthetes such that brighter color is associated with smaller numerical size (Kadosh et al., 2005) and physical size (Walker and Walker, 2012). The similarity of size–brightness mapping for both abstract (i.e., numbers) and concrete (i.e., physical objects) size is consistent with these effects arising from a higher-level conceptual stage.

Finally, the relationship between vision and touch also supports a conceptual-level relationship that underlies both synesthetic and non-synesthetic mappings. Two recent studies reported that non-synesthetic participants matched haptic sensations to visual features in ways akin to an individual with tactile–visual synesthesia. Specifically Simner and Ludwig (2012; also see Ludwig and Simner, 2012) studied EB, who experiences synesthetic colors from tactile stimuli. They found that, for EB, smooth, soft, and round stimuli tended to induce brighter colors, compared to rough, hard, and spiky stimuli. Similar patterns between touch and vision were also found in non-synesthetic participants, despite them having no conscious color experiences. Moreover, for both EB and non-synesthetes, some tactile stimuli seemed to form a reliable link with certain colors (e.g., soft stimuli were associated with pink). The authors interpreted the resemblance as stemming from shared mechanisms, such as semantic knowledge. For example, the link between softness and pink might originate from the Western convention of pairing pink color with infancy and femininity, two traits indirectly associated with softness. Thus, as with other types of synesthesia, the similarity in underlying patterns between tactile–visual synesthetes and non-synesthetic individuals is consistent with the involvement of high-level cognitive processes at a conceptual stage.

The studies reviewed above suggest that synesthesia relies on mechanisms involving ordinary semantic representations and abstract concepts, echoing the view that synesthesia may be an exaggerated form of “normal” cognitive operations (Martino and Marks, 2001; Ward et al., 2006). In particular, the knowledge regarding lexicon–color and cross-modality association seems to mediate the “content” of synesthetic experience (e.g., its hue or brightness). This suggests that the link between synesthetic inducer and concurrent is not random and arbitrary but guided by conceptual knowledge about lexical/semantic properties and cross-modality relations. In the following section, we review more evidence showing that conceptual mediation seems a general principle for other types of synesthesia.

### IS CONCEPTUAL MEDIATION A GENERAL MECHANISM FOR SYNESTHESIA?

As discussed earlier, it has been found that most grapheme–color synesthetes report experiencing the same synesthetic color regardless of the modality (printed or spoken) and format (italic, bold, or different fonts) in which the inducing stimulus is presented (Rich et al., 2005). In addition, it has also been found that an ambiguous graphemic stimulus can induce different synesthetic colors when interpreted differently (e.g., an ambiguous symbol can be interpreted as “S” or “5”; Myles et al., 2003; Dixon et al., 2006). These findings suggest that grapheme–color synesthesia relies crucially on the concept of lexical meaning. There are also some recent reports of novel forms of synesthesia that are completely conceptual in nature. Nikolić (2011) documented two individuals who report that different swimming styles (breaststroke, butterfly, backstroke, and front crawl) evoke different colors. This “swimming-style synesthesia” is determined by conceptual rather than perceptual factors: the same color is elicited no matter whether the synesthete is receiving proprioceptive feedback during swimming, performing motor imagery of swimming action, or even just seeing the photos of swimming posture. The determinant of color seems to be the concept of swimming style with little impact of perceptual factors. Such observations led the authors to dub the phenomenon “*ideasthesia*” and propose that it is the “idea” of the inducer that triggers synesthetic colors (Jürgens and Nikolić, 2012; Mroczko-Wasowicz and Werning, 2012).

The claim that conceptual mediation is critical for synesthesia seems incompatible with a recent study showing that letters with similar shapes evoke similar synesthetic colors (Brang et al., 2011). These authors interpreted this finding as neurons coding graphemic features activating those coding chromatic sensations in the contiguous V4, hence synesthetic color being an outcome of low-level neural wiring. Another recent study, however, showed that exactly the same result could be explained by high-level conceptual mechanisms: Jürgens and Nikolić (2012) trained 20 German synesthetes to learn artificially created graphemic symbols for 10–15 min and asked them to report whether these symbols evoked colors after training. Results of subjective reports showed that these pseudo graphemes were able to provoke color experience (also see Mroczko et al., 2009 in which the authors demonstrated rapid transfer of synesthetic colors to novel symbols using an objective congruency effect in a color naming task). Critically, these new “graphemes” tended to “inherit” synesthetic colors from German letters that were similar in shape. As it only took minutes to form the novel associations between “graphemes” and colors, the authors suggest that such a timeframe would be too rapid for the formation of new neural cross-wiring to occur. Instead, the result is more likely to be driven by high-level conceptual processing that guides the choices and creation of synesthetic colors. In our view, this implies similar mechanisms may operate during language acquisition in early childhood such that a synesthete may learn a new grapheme based on its visual similarity to a grapheme learnt earlier, which consequently affects its synesthetic color. For example, if “O” is learnt first and associated with lime green, “Q” may be initially learnt as “O with a stroke” and inherit the synesthetic color from “O”; as “Q” gradually develops its graphemic individuality, its lime green color may turn into another color with similar hue, such as olive green. Although speculative, this conceptual account may also explain why similarly shaped letters of the first and second language tend to induce similar colors (Rich et al., 2005; Barnett et al., 2009).

In addition to the types of synesthesia reviewed above, concepts have also been found to mediate “lexical-gustatory synesthesia,” in which words are associated with tastes (e.g., the word “castanets” elicits the taste of tuna). In an elegant demonstration, Simner and Ward (2006) presented images of objects that are rarely encountered in daily life (e.g., a platypus) and had six lexical-gustatory synesthetes perform picture naming. As the objects were uncommon, sometimes participants experienced the “tip-of-tongue” (TOT) phenomenon in which they recognized the object and knew many of its properties but just failed to retrieve the name. When TOT occurred, the experimenter then questioned the participant further about the word’s phonology (to ensure that the participant could not retrieve any information about constituent letters or the number of syllables) and asked synesthetes to report the synesthetic taste. After the naming task, the experimenter read aloud the names of objects and asked synesthetes to indicate their synesthetic tastes again. The key result was that in the state of complete TOT (i.e., neither name nor any phonological or lexical piece could be retrieved) synesthetes sometimes still reported experiencing tastes, and the reported tastes matched with the tastes they had subsequently when hearing the objects’ names. Simner and Ward (2006) suggest that thinking of a word’s meaning is sufficient to trigger synesthetic tastes, occurring even in the absence of access to any lexical or phonological code. Together with other types of synesthesia, the overall pattern of evidence seems to indicate that the key stage of the information processing stream at which synesthetic percepts arise is the activation of concept. By contrast, stages upstream (e.g., perceptual analysis) or downstream (e.g., lemma or phonology) to the concept/meaning of the inducer do not seem essential for the elicitation of synesthetic experiences.

Having considered the behavioral evidence showing the key role of conceptual knowledge in mediating synesthesia, in the next section we turn to the brain, discussing what neural structures are potential substrates for conceptual mediation. We focus especially on the neural representations of object color knowledge, and the predictions our conceptual-mediation model makes for the regions involved in synesthesia beyond V4.

### THE ROLE OF THE ANTERIOR TEMPORAL LOBES IN REPRESENTING OBJECT COLOR KNOWLEDGE AND GRAPHEME–COLOR SYNESTHESIA

**FIGURE 3:**
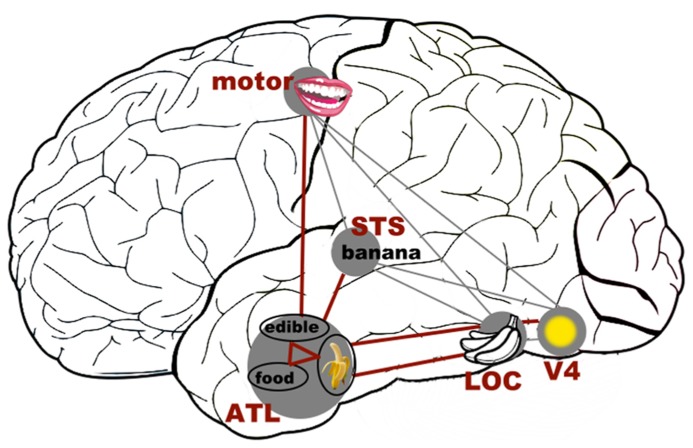
**A schematic representation of how the (much simplified) concept of a banana as an edible food with its distinct color and shape could be represented in the “hub-and-spoke” framework** (Patterson et al., 2007; Lambon Ralph and Patterson, 2008). In this model, various perceptually based attributes are coded in their respective modality-specific regions (spokes) for perception and action (i.e., color: V4; shape: lateral occipital cortex, LOC; associated action of biting: motor-related regions lying along the precentral gyrus; linguistic label: superior temporal sulcus, STS). There may be interconnections among these regions, as indexed by gray thin lines. Crucially, these attributes are connected to (shown as thick maroon lines), and communicate through, an amodal (or supramodal) “hub” in the (possibly bilateral) anterior temporal lobes (ATLs). At the hub stage, different sources of sensory and motoric features coalesce to form abstract meaning. Note that this is a simplified representation of the typically intricate connections of a semantic network.

There is good evidence that the anterior temporal lobe (ATL) serves as a core substrate in the neural architecture for conceptual/semantic knowledge (Patterson et al., 2007; for review, also see Lambon Ralph and Patterson, 2008). Various lines of inquiry using neuropsychological and neuroimaging approaches have shown that the ATL mediates conceptual processing for a variety of stimuli (words, images, sounds, odors, etc.). This has led to the “hub-and-spoke” model of conceptual representation, where the ATL acts as a “hub” that works in tandem with modality-specific areas (spokes) to integrate various perceptually based content into a meaningful conceptual entity (Patterson et al., 2007; Lambon Ralph and Patterson, 2008). As illustrated in **Figure 3**, for example, the concept of a banana as an edible food that is yellow in color and curvy in shape requires the concerted operation of modality-based regions processing color, shape, motor information, and the ATL constructing more abstract concepts about “banana,” “edibility,” and “food.” One fMRI study of synesthesia and a recent neurostimulation study of ordinary color knowledge together give hints that synesthetic color experience may recruit a portion of the ATL that also underpins typical conceptual memory of object–color associations. This preliminary neural evidence, in combination with the previously reviewed behavioral results showing that synesthetic color resembles color memory and relies upon semantic representation, lead us to speculate that there is a role for the ATL in the neural substrate of synesthesia.

Could the ATL be a key neural structure in synesthesia? We propose that, based on the behavioral evidence of conceptual processing being important in synesthesia, and on the role the ATL plays in normal object knowledge, it could be a key component of the neural substrates that give rise to synesthesia. At this point, there are a few hints in the published literature and recent work from our own laboratory supporting such a speculation. First, van Leeuwen et al. (2010) reported an fMRI exploration in which, in one of many analyses, synesthetic color appeared to recruit a portion of the right ATL. In an initial experiment, these researchers analyzed brain activation in 19 synesthetes and 19 controls within an occipitotemporal ROI to identify areas that were sensitive to real and synesthetic colors. In a second experiment, they then tested the two groups using a repetition suppression experiment to explore where neurons responded to actual color (i.e., a physically colored square; target) as if it was the same as a previously presented synesthetic color (i.e., a color-inducing achromatic grapheme; prime). The authors hypothesized that there would be a drop in neural activation in the color-sensitive areas (V4, delimited based on the localization results from the first experiment) when the prime (synesthetic color) and the following target (real color) were of the same hue relative to different hues. The predicted effect would mean that synesthetic colors are coded by the color center V4, but instead the results showed *no* suppression effect of synesthetic color in any of the pre-defined color-sensitive ROIs of the visual cortex. Subsequent analyses of brain regions outside the color-selective ROIs, however, found repetition suppression in synesthetes in the right ATL and the hippocampus, two areas crucial for memory formation and conceptual knowledge (Tulving and Markowitsch, 1998; Lambon Ralph et al., 2009). The authors concluded that synesthetic color does not fully share a neural basis with real color and is represented by high-level cortical areas beyond the scope of the visual cortex, such as those involved in memory. Note, however, that the neural suppression effect of synesthesia was obtained using planned tests in the absence of a significant interaction involving the group factor (synesthetes vs. controls). Such results must therefore be treated cautiously, but they do provide tantalizing hints that synesthetic color may be represented in brain areas involved in high-level functions of semantic memory.

The involvement of the ATL in representing conceptual knowledge (Patterson et al., 2007), and the possible repetition suppression effect in this region of synesthetic brains (van Leeuwen et al., 2010), give an (admittedly preliminary) basis for a plausible cognitive-neural model in which synesthesia relies on “normal” mechanisms for conceptual knowledge. A critical step for such a model is first to demonstrate that this region mediates the conceptual associations between objects and canonical colors in non-synesthetes. We recently tested this hypothesis by disrupting the neural processing in the ATL using continuous theta-burst stimulation (cTBS, a high-frequency form of transcranial magnetic stimulation, TMS) to explore whether the ATL is necessary for integrating typical color with object shape and name (Chiou et al., in press). In separate experimental sessions, we disrupted neural processing of either the left ATL or a control site (the occipital pole) in eight non-synesthetic participants. In each session, participants named images of different objects with characteristic colors; the objects were presented in either their typical or an atypical color, which results in a color congruency effect (e.g., faster object recognition/naming of a yellow banana than a purple banana). Participants also performed a control task that required naming the amount of numbers in an array, where the element digit could be congruent or incongruent with the total amount (e.g., 333 or 555). This task produces a congruency effect that does not involve conceptual integration but is driven by conflicts in lexical retrieval. The critical result is that ATL stimulation abolished the otherwise robust color congruency effect but had no impact on the numerical effect, indicating a selective disruption of object color knowledge. Neither color nor numerical congruency effects were affected by stimulation at the control occipital site, ruling out alternative explanations of TMS disrupting any congruency-type effect in a non-specific manner. This result suggests that the ATL plays a key role in mediating the coupling between objects and their canonical colors.

We speculate that the neural computation performed by the ATL to link object concepts to canonical colors may be analogous to that involved in linking inducer concepts to synesthetic colors. When synesthetic color becomes strongly bound to the concept of an inducer (e.g., letter), it becomes a feature or attribute of that inducer, which shares characteristics with typical conceptual knowledge about semantics and cross-modality relations. This would explain why synesthesia is unaffected by early perceptual mechanisms (e.g., brightness contrast, chromatic adaptation, etc.), shares systematic patterns with normal cross-modality correspondences, and is influenced by high-order cognitive factors (e.g., identification of meaning, contextual interpretation, lexical/semantic relationship with other conceptual entities, etc.).

If the ATL is crucial for synesthesia, why have we not seen activation in this region in neuroimaging studies of synesthesia? There are clear methodological reasons why this may be the case. First, a recent meta-analysis has suggested that the absence of the ATL activation may reflect technical limitations of magnetic resonance imaging (Visser et al., 2010b). Specifically, images of the ATL are usually distorted due to field inhomogeneity around the air-filled cavities near the ATL (Devlin et al., 2000), hence leading to substantial signal dropout. In addition, many neuroimaging studies have limited coverage of the temporal lobe due to a restricted field of view (FOV) during data acquisition. According to Visser et al. (2010b), the inferior section of the ATL is often not included when the FOV is narrower than 15 cm. The second major contributing factor to the lack of ATL activation is the prevalent use of ROI approach: with the aim to test whether synesthesia activates color-sensitive area V4, and to maximize power in the often small samples, it is common for synesthesia studies to confine the analyses to the ventral occipitotemporal areas, ignoring other brain regions. Together, the lack of ATL activation is likely to reflect an absence of evidence rather than an evidence of absence. Some recent papers have shown that with distortion-corrected techniques that prevent signals of the ATL from degradation, ATL activity is reliably observed when participants retrieve conceptual knowledge (Binney et al., 2010; Visser et al., 2010a; Visser and Lambon Ralph, 2011). We therefore may yet see studies demonstrating the involvement of the ATL in synesthesia if the technical issues concerning dropout are taken into account in experimental design and data acquisition.

It is worth noting that the possible repetition suppression reported by van Leeuwen et al. (2010) was in the *right* ATL, whereas Chiou et al. (in press) stimulated the *left* ATL. There is evidence showing that the bilateral ATLs contribute equally to conceptual processing, no matter whether the stimuli are graphemic or pictorial (Lambon Ralph et al., 2009; Pobric et al., 2010; but see Gainotti, 2012). It is possible that TMS to the left ATL can generate some subtle effects on the right ATL, through connections to homologous regions (e.g., Schambra et al., 2003; although note that here the contralateral effect is based on the excitability of the motor cortex, whether this is generalizable to the ATL remains unknown). Alternatively, there may be a hemispherical difference between the left and right ATL in representing synesthetic color and object color knowledge.

It is important to stress that we are *not* suggesting that synesthetic experience is merely conceptual association, mental imagery, or metaphoric thinking. Synesthesia is elicited involuntarily by a recognized inducer and is experienced as a conscious percept, two diagnostic features making it distinct from voluntary imagery and ordinary conceptual knowledge. Nonetheless, the psychophysical similarity between synesthetic color and object color memory, alongside the possible involvement of the concept-related ATLs, suggest that the ostensibly perceptual synesthetic experience requires critical non-perceptual mechanisms linking inducers and concurrents. Additional mechanisms, however, may still be necessary to enable a synesthetic link between inducers and concurrents to emerge as a conscious percept in a person’s subjective experience. The key mechanisms that make synesthetic experience less intentional than, and thus distinct from, other concept-related phenomena (e.g., cross-modal mappings, typical color knowledge, etc.) may lie in the broad neural structures that subserve attention and feature integration, including the parietal lobe (Taylor, 2001; Laureys, 2005). Exploring the dynamics between the ATLs and parietal regions will be crucial for developing our neurocognitive conceptual-mediation model of synesthesia. In the following section, we raise some testable predictions.

### SOME TESTABLE HYPOTHESES REGARDING THE ROLE OF THE ATL IN SYNESTHESIA

**FIGURE 4:**
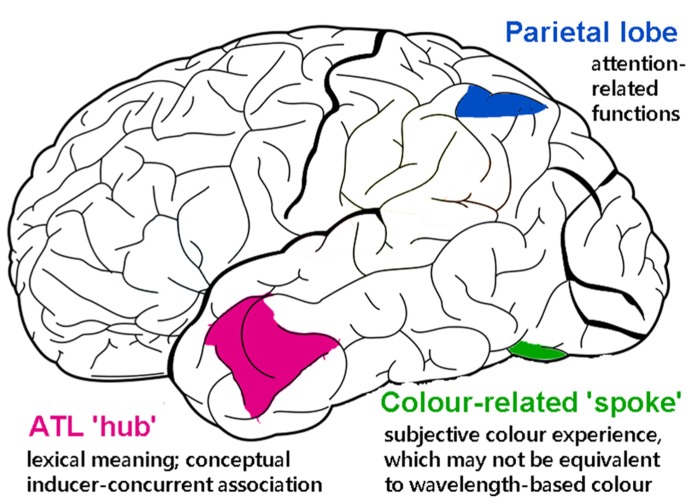
**The outer cortical surface with proposed regions involved in the neural substrates of grapheme–color synesthesia under the conceptual-mediation model.** The *anterior temporal lobe (ATL)* is indicated in magenta. In our model, the ATL functions as a representational “hub” that processes lexical meaning and associates the lexicon to synesthetic color representation at an abstract, conceptual level. The *color-related area* of the posterior ventral temporal cortex is indicated in green. This cortical region contains the fusiform gyrus (V4) and the lingual gyrus. In our model, this area serves as a “spoke” that processes the subjective experience of synesthetic color and may not overlap with the neural basis of real color experience. The *posterior parietal lobe* is indicated in blue. This region mediates the attention-related functions in synesthesia, such as directing voluntary attention to an inducer or binding graphemes with color at a perceptual level, akin to its role in the typical function of binding various visual attributes (color, shape, location, etc.) of an object.

We propose that the ATL is a critical node in the neural network underpinning the conceptual mediation observed in synesthesia (see **Figure 4**), reflecting the accumulating evidence of the importance of identification of meaning and conceptual modulation. The ATL serves as a “hub” that mediates conceptual integration between inducer meaning and its associated concurrent. After the sensory cortices perform the initial analysis of the inducer’s perceptual properties (e.g., visual word form or acoustic features of a sound), the processing stream feeds into the ATL, which functions as a repository for a myriad of high-level conceptual attributes. Neural activity in the ATL underpins the conceptual entity of the inducer and allows access to associated conceptual attributes. Analogous to its role in the ordinary conceptual integration between objects and typical colors (Rogers et al., 2007; Hoffman et al., 2012; also see Chiou et al., in press), we propose that the ATL also mediates the connection between synesthetic inducers and concurrents. Note that although the outcome of this connection is anomalous (i.e., a conscious experience not present in non-synesthetes), weak versions of this link presumably also exist in non-synesthetes, leading to implicit associations.

It should be emphasized that the ATL performs the integration at a conceptual (or supramodal) level. Namely, it combines the meaning of the inducer with an abstract concept of the concurrent that may be devoid of perceptual content (e.g., letter “A” is red in color or middle C is metallic gray – linking perhaps in a propositional/symbolic manner). To gain access to the embodied/phenomenological content of synesthetic experiences, the ATL “hub” needs to work in tandem with cortical regions (spokes) that are adjacent to (but may not overlap with) those subserving perceptual features, hence a distributed coding pattern that involves (at least) dual sites. In the case of synesthetic color, for example, the “spoke” may be the *left medial lingual gyrus*, an area close to the typical anatomical locus of human V4 and has been reported to be activated by retrieval of object color knowledge (Chao and Martin, 1999) and synesthetic color (Rich et al., 2006). Although synesthetic color may recruit “spoke” areas of the broad perceptual cortices (e.g., the lingual gyrus), it does not necessarily require this to be the same core cortical sites as those coding the wavelength of real color (e.g., V4/the posterior fusiform gyrus), which could explain the documented differences between actual and synesthetic colors. That is, the “spoke” representation of synesthetic color may be coded in the *vicinity* of but does *not* *necessarily* *overlap* with V4. Furthermore, we suspect that the individual variability in the salience of synesthetic color may lie in the “strength” with which the ATL “hub” connects with the color-related “spoke” (i.e., how far and persistently the neural activation travels). In synesthetes who *see* vivid lexically induced colors, there may be reliable feedback signals of the ATL reaching to and activating the broad color-related visual cortices. By contrast, in synesthetes who *feel* color, the neural activation may not perpetuate through to the perceptual cortices – they may even stay within the scope of the ATL. Note these are just examples; the range of synesthetic experience seems to us to be a continuum, not a dichotomy.

In addition to the “hub-and-spoke” system that amalgamates inducers with concurrents, the elicitation of synesthetic experiences also requires the parietal lobe. Based on the strong involvement of the posterior parietal lobe in attention (Donner et al., 2002), its contribution to synesthesia is typically interpreted as a facilitation of attentional binding between grapheme and color (Esterman et al., 2006; Muggleton et al., 2007). However, there are two possible (not mutually exclusive) roles for the parietal lobe in synesthesia: first, it has a domain-general function in that it works as a part of the fronto-parietal cognitive control system that prioritizes stimuli and directs voluntary attention to an entity pertinent to synesthetic experience, such as a color-inducing digit. Second, it has a domain-specific function that it acts as “glue” that binds inducer and concurrent into a united whole. The second function of the parietal lobe may convert the content of a synesthetic concurrent into a conscious experience, making it more vivid than ordinary conceptual knowledge of color.

The strength of the model outlined above is that it generates specific predictions and is testable (and thus falsifiable). A straightforward prediction is that disrupting the neuronal activity of the ATL will lead to a breakdown of the conceptual coupling between synesthetic inducers and concurrents. This disruptive effect should occur in any type of synesthesia that involves conceptual association. Therefore, although auditory–visual and grapheme–color synesthesia involve different inducing stimuli and recruit distinct brain regions for initial perceptual processing, TMS to the ATL should disrupt both types of synesthesia if they both rely on the conceptual integration executed by the ATL. It is noteworthy that this predicted TMS effect of the ATL might disrupt distinct processes from the disruption caused by TMS to posterior parietal regions. Whereas TMS to the parietal areas may impair the attentional processes involved in conscious synesthetic percepts, TMS to the ATL should cause disruption at a stage prior to the involvement of parietal mechanisms – the conceptual coding of inducers and their conceptual link to concurrents.

## CONCLUDING REMARKS

In this article, we review evidence demonstrating the critical contribution of conceptual representations in synesthesia. There are some claims that synesthesia may involve low-level visual mechanisms and acts like the color experience caused by the wavelength of light. The weight of evidence, however, suggests that synesthesia relies on high-level cognitive processes, such as identification of lexical meaning, and behaves distinctly from real color in terms of its psychophysical properties. Furthermore, neuroimaging investigations have failed to find consistent activation of the core cortical substrate of color vision (V4) when an individual experiences synesthetic color, consistent with the suggestion that cortical areas beyond the scope of the early perceptual system may be crucial. We suggest that the data call for a re-evaluation of the current neurocognitive models of synesthesia, which assume that synesthesia is a perceptual phenomenon. Based on recent behavioral and neural evidence, we speculate that the synesthetic mapping between inducers and concurrents may be analogous to the typical conceptual mapping between objects and their perceptual attributes. This conceptualization of synesthesia is compatible with recent reports that synesthetic color experience is akin to color memory. It also better explains the strikingly similar trends of non-synesthetic individuals to synesthetes. In a broader sense, we believe this framework can potentially inform us how the high-level cognitive system shapes our conscious experience of the world.

## Conflict of Interest Statement

The authors declare that the research was conducted in the absence of any commercial or financial relationships that could be construed as a potential conflict of interest.

## Footnotes

1 van Leeuwen et al. (2010) categorized 21 synesthetes into associators or projectors using a questionnaire. In their fMRI experiments, none of their region of interest analyses revealed a reliable difference between the two groups. Although they reported associators showed “marginally” higher (*p* = 0.09) activation in a left parietal lobule cluster than did projectors, these effects appeared rather weak as they were obtained using planned tests (due to null results of their main analyses) with liberal criteria (i.e., α = 0.05, not corrected for multiple comparisons).

2 Although Hubbard et al. (2005) draw a parallel between their “higher/lower” synesthetes and the “associator/projector” distinction discussed previously, Ward et al. (2007) later found these categorizations to be orthogonal.
